# Extraction of Steel from the Interior of the Eye with the Electro-Magnet1Read before the New York State Medical Association, November 15, 1892.

**Published:** 1893-02

**Authors:** Alvin A. Hubbell

**Affiliations:** Buffalo, N. Y.; Professor of Ophthalmology in the Medical Department of Niagara University; Surgeon to the Charity Eye, Ear, and Throat Hospital, etc.; 212 Franklin Street


Buffalo Medical and Surgical Journal

VOL. XXXII. FEBRUARY, 1893. No. 7.

### Original Communications.

### EXTRACTION OF STEEL FROM THE INTERIOR OF THE EYE WITH THE ELECTRO-MAGNET.[1]

BY ALVIN A. HUBBELL, M. D., Buffalo. N. Y.

Professor of Ophthalmology in the Medical Department of Niagara University; Surgeon to the Charity Eye, Ear, and Throat Hospital, etc.

THE use of the magnet in removing steel from the eye has been suggested and practised at various times in the past, but the merit of effectually introducing it and stimulating the profession to examine into its utility is due, first, to Dr. W. A. McKeown, of Belfast, Ireland, who, in 1874, published (*British Medical Journal*, Vol. I., 1874,) his experience with the permanent magnet, constructed with a tapering point, and to Dr. J. Hirschberg, of Berlin, who, in 1881, published (*Archives of Ophthalmology*, Vol. X., 1881,) the results of his experiences with the electro-magnet as most ingeniously adapted to the eye by himself. Since then various forms of both permanent and electro-magnets have been constructed, and every good ophthalmic surgeon makes one of these instruments an especial part of his armamentarium. The permanent magnet, however, has so little power of attracting iron as compared with the electro-magnet that the latter has quite superseded the former. Hirschberg's electro-magnet is, perhaps, the form most used at the present time, yet, of all with which I am acquainted, this is the most cumbersome and unwieldy.

It was upon the failure of a Hirschberg magnet in the hands of a friend, to serve the purposes desired, together with peculiar circumstances pressing upon me, and a desire to save a patient's only eye, that I was led, in 1884, to devise an instrument that seemed to me to be more in accord with the needs of the case then put into my hands. I submitted the plan for an electro-magnet to a

1. Read before the New York State Medical Association, November 15, 1892.

practical electrician of my city, who made for me the one heretofore published in the BUFFALO MEDICAL AND SURGICAL JOURNAL for July, 1888. George Tiemann & Co., of New York, have, at my suggestion, recently made some improvements in its construction, preserving at the same time the essential features which distinguished the original.

The advantages claimed for this magnet are: Its great power of attraction, its lightness, its small size, and its shape, most convenient for manipulation. With this instrument I have extracted steel from the interior of the eye in the following cases:[1]

CASE I.—M. S., aged 43, boiler-maker, was struck in his left eye June 18, 1884. The sight, he says, was destroyed at once, and an ophthalmic surgeon enucleated the ball.

On the morning of the 27th of August following, two months after the accident to the left eye, while striking a "set," a chip of steel flew into the right eye. The eye "watered" some afterwards, but there was very little pain, and the vision was "pretty good." Attempts were made on the 28th by a fellow-practitioner to extract the steel through the wound produced by it, by means of a Hirschberg magnet, but without success. The doctor declined to make further efforts to get the foreign body, and the patient was placed in my hands on the evening of August 30th. The eye at this time was in constant pain, with a "pricking" sensation in its upper part. Vision was reduced to counting fingers. There was considerable lachrymation, the lids were somewhat swelled, the eyeball was very red, and the conjunctiva of the lower part was chemotic. There was a wound two to three millimeters long at the junction of the lower part of the cornea and sclera. This was still open and a bead of vitreous was pressing through it. The pupil was irregular in shape, a piece of iris evidently having been cut off in the direction of the wound. The ophthalmoscope showed the fundus to be greatly obscured by hemorrhage or inflammatory products in the vitreous, and the steel could not be seen. I was assured, however, that it had been seen in the vitreous. The lens appeared to be perfectly transparent, the wound and field of previous operations having been through the suspensory ligament.

The case did not present a very hopeful prospect, but as this was the only eye left to him, I was willing to add my efforts to those that had already been made to extract the steel, and, if possible, save at least a little vision. The first thing needful, however, was a good electro-magnet, and this I did not possess, and my only means of obtaining one within a brief time was to have one made. I at once applied to an electrician of my city, and suggested to him how one, in my opinion, should be made. At 10 o'clock the next day he had one ready.

At 11 o'clock A. M., August 31st, just four days after the injury,

1. Cases I. and II. have already been published in the JOURNAL in July, 1888.

I proceeded with the proposed operation. The patient being anesthetized, I dissected back a small triangular flap of conjunctiva from the sclera, between the equator of the ball and the ciliary region, and between the external and inferior and recti muscles. After the hemorrhage was stopped I made an antero-posterior incision through the sclera into the vitreous. This incision was about three millimeters long. A few drops of straw-colored fluid escaped. Through this incision I introduced into the vitreous an extension-point of the electro-magnet, about one centimeter in length and one millimeter in diameter at its end. I directed the point at first towards the center of the ball and posteriorly, but found nothing. I then directed it forwards, when I both felt and heard a distinct click, which was also heard by the bystanders. On withdrawing the point the steel came with it, firmly held in its magnetic grasp. It was a thin scale about three millimeters in length and one and a half in width. The conjunctival flap was replaced over the incision and sutured at its apex.

Very little reaction followed the operation, and both the original and operative wounds healed kindly. After several months the opacities had cleared up in the vitreous, and the patient was able to read No. 2 Jaeger, at fourteen inches, with the aid of + 1.00 D. glass. The visal field was considerably contracted—most, however, on the inner side. The ophthalmoscope showed marked choroidal changes, such as are found after choroiditis, and these were numerous, but most marked in the lower and outer parts of the fundus. The patient reads a great deal and is enabled to earn an independent livelihood.

Case II.—April 5, 1888, I was invited by Dr. Frank W. Abbott, of Buffalo, to assist him in the following case: On April 3d, Miss A., about 20 years of age, was stitching leather with a sewing machine, when the needle broke and a piece struck her right eye. She applied to Dr. Abbott for advice, not, however, suffering much distress or pain. On examination, there was scarcely any evidence of injury, except that the anterior chamber was obscured with blood. A solution of atropia was instilled, and she was asked to return in two days. At this time I was invited to see the case. The blood had become absorbed, and the pupil was nearly at its maximum dilatation, thus permitting an easy examination of the fundus. The ophthalmoscope showed all the media to be transparent. A piece of the sewing-machine needle could be distinctly seen in the vitreous, projecting from the anterior and inner part of the cavity directly towards its center. A slight mark could be distinguished externally at the inner corneo-scleral junction, indicating the point of its entrance. It had evidently passed through the iris, and nearly through the ciliary body, by which it was now firmly held. The eye was slightly red and irritable.

Arrangements were made for an operation on April 6th, at which I assisted with my electro-magnet. The patient being anesthetized,

Dr. Abbott excised a piece of conjunctiva immediately posterior to the point of entrance of the needle, on the inner side of the ball, over the ciliary region. The hemorrhage was stopped, first using pieces of ice, but afterwards cotton steeped in hot water, which was much more effective. He then made an antero-posterior incision directly down to the steel through the sclera and ciliary body into the vitreous cavity, about three millimeters long. The magnet being in readiness, I introduced a small extension through the incision, and it at once grasped the broken needle, but without loosening it. I held it steadily with the magnet while the doctor enlarged the incision anteriorly until it was entirely disentangled, when it was easily withdrawn, adherent to the magnet.

The conjunctival wound was closed by two sutures passed perpendicularly to the sclerotic incision. The patient was kept quietly in bed a few days with the eye bandaged, and atropia was occasionally instilled.

I examined the eye four weeks after the operation, and it appeared to have fully recovered, and vision was, the patient said, "as good as ever."

Case III.—Patrick H. E., aged 28, boiler-maker, while caulking a boiler, using a hammer and caulking tool, on August 6, 1888, was struck by a scale of iron on the outer part of the cornea of the left eye. He visited an oculist of Buffalo, who examined his eye and told him that there was steel in it. He prescribed for him a two-grain solution of atropia, to be instilled every two hours, and directed him to call in two days. The patient then went to his family physician, who advised him not to wait so long, and sent him to me. I found a vertical wound in the left cornea, one millimeter to the inside of the outer margin, and two millimeters long. The iris beneath also had a vertical wound a little shorter and slightly gaping. No foreign body could be seen by an external examination, and with the ophthalmoscope the fundus appeared clear posteriorly; but to its outer side there was a dark, opaque spot behind the region where the iris was wounded. The vision was normal. There was no pain in the eye, but there was some lachrymation, and the eyeball was reddened. I diagnosed steel in the eye and proceeded to find and remove it, if possible, with the electro-magnet. I used a small extension point, a little less than one centimeter in length. After thoroughly cocainizing the eye I introduced this through the wound of the cornea and iris, produced by the foreign body, carefully avoiding the crystalline lens. I passed the point to the depth of about one-half centimeter, when the iron struck it with a distinct click, which was heard by all present. It was firmly held by the magnet, and was drawn out through the wound by a little effort and after enlarging the wound slightly with a knife, the edges of the steel being ragged and catching on the surrounding tissues. The magnet was aided by forceps in the extraction, after the steel was brought to the wound.

The steel was a thin scale seven millimeters long and two millimeters wide at its widest part, and weighed one grain. The crystalline lens did not seem to have been wounded, either by the passing in of the steel or its removal. The eye was dressed with 1 to 4,000 bichloride solution, and a four-grain solution of atropia ordered to be used every two hours. The eye was considerably inflamed for two days after the operation, but from that time on recovered rapidly, and on September 29th it was well. There was a slight scar of the cornea, and the iris showed a vertical slit at the point of injury. The crystalline lens was dotted with opacities. The vision equaled No. 60 Snellen at four meters, and Jaeger No. 18, slowly, at ten inches. The patient was not seen afterwards. In this case the eye was saved, but a traumatic cataract followed, which, if successfully removed later, would undoubtedly have given good vision.

Case IV.—George C., aged 29, blacksmith, consulted me on the evening of August 6, 1890, for an injury of his eye. That afternoon, while cutting a bolt with a "cold-chisel," a piece of the steel flew off and struck his left eye. I found a vertical wound about four millimeters long, just outside of the inner cornea-scleral junction, and the iris was protruding through it. The anterior chamber was filled with blood and the fundus of the eye could not be seen with the ophthalmoscope. No foreign body could be seen upon external examination, but I was of the opinion that a piece of iron had entered the vitreous space. Under cocaine anesthesia, I cut off the protruding iris, and with the electro-magnet, armed with an extension-point, one centimeter in length and one and a half millimeters in diameter at its end, I began my search. On introducing the point to a depth of about one-half centimeter, it caught the iron with a pronounced click, and on being withdrawn brought it to the corneal wound. I found it to be a large piece of irregular and ragged contour, and it became necessary to enlarge the wound a little and assist with forceps before it could be drawn out. The conjunctiva was drawn together over the sclerotic wound by a suture. After instilling a six-grain solution of atropia, the eye was dressed with 1 to 4,000 bichloride solution, and both eyes were bandaged. The piece of steel was one centimeter long, one-half centimeter wide, and two millimeters in thickness, and weighed seven grains. Some inflammatory reaction followed the operation, but this soon subsided. The pupil was kept dilated by atropia and the suture was removed on the fourth day. The patient was discharged September 29th, with vision equaling No. 24 Snellen, at three meters. At this time the media were all clear, but the fundus showed several spots of choroidal atrophy at its inner side.

Case V.—Michael B., aged 39, laborer, consulted me December 13, 1890. Two days previously a piece of steel from a hammer struck his right eye. The eye pained him for a short time, but has since been easy, and at this time was only slightly red. Vision equaled No. 60 Snellen, at one meter. The anterior chamber was normal; pupil normal in size and responded well to light. At the outer part of the cornea, one millimeter inside of its margin, there was a vertical cut, one millimeter in length, nearly healed. The iris showed a minute vertical opening at its outer side, half way between its pupillary margin and periphery, the bottom of which appeared whitish. The pupil dilated readily under cocaine, and the ophthalmoscope showed the lens to be slightly hazy at its posterior part, and at its outer and anterior portion was a milky-looking reflection, one millimeter wide, starting from the iris-wound and running backwards about four millimeters. Instillations of atropia and boracic acid solution were used every four hours.

December 14th, 9 A. M., consultation was held with Dr. Abbott, who agreed with me that there was steel in the eye. Under cocaine I made a vertical incision of the cornea near its outer margin and over the iris-wound, and introduced through this and the opening in the iris electro-magnet points of different sizes and lengths, but could not find the steel, although they were passed deeply into the vitreous as far as seemed prudent. The eye was then dressed, and atropia and boracic acid solution ordered as before.

December 16th, the eye was painful and much inflamed. Gave the patient chloroform and made another attempt to find the steel with the magnet, and succeeded, introducing the point through the same wound as before. The steel was very minute in size, being a thin scale, two millimeters in length and half a millimeter in width. Inflammation, which had already begun, continued and resulted in panopthalmitis, necessitating enucleation of the ball at the end of four weeks.

Case VI.—Fred T., aged 39, laborer, visited me December 5, 1891. He stated that the day before, while cutting off a rivet from a salt evaporating-pan with a chisel, he was struck in the right eye by a piece of iron. Upon examination of the eye I found it inflamed, with a wound in the ciliary region near the outer border of the cornea, extending obliquely downwards and inwards, and about six millimeters in length. The iris was protruding between the lips of the wound, and both the aqueous and vitreous chambers were filled with blood. Vision equaled no perception of light. Under cocaine the iris protrusion was cut off. An exploration was then made with the electro-magnet, using an extension-point, one centimeter long and two millimeters in diameter at its end. On passing the magnet about three millimeters into the vitreous and directing it backwards, some stagnant blood flowed out and the iron was attracted to the magnet with a pronounced click, as is usual in such cases. The piece of iron seemed to lie across the wound, and it was worked down and back with a silver probe, and after a little came out end-wise without enlarging the wound. I instilled a four-grain solution of atropia and dressed the eye with 1 to 4,000 bichloride solution.

The iron was a thick scale, twelve millimeters long by four wide, and weighed three grains. Considerable inflammation followed, the conjunctiva becoming chemotic and the eyelids swelled. This subsided, however, in a few days, when he returned to his home, fifty miles distant, after which I lost sight of him.

Case VII.—William M., aged 22, boiler-maker, called at my office November 30, 1891, and stated that he had been struck in the left eye about an hour previously with something while caulking a boiler with a hammer and caulking tool. I found some blood in the lower part of the anterior chamber, and there was a cut through the sclera, just below and to the inner side of the cornea, extending horizontally inwards, and about three millimeters in length. No foreign body could be seen by .external examination. The pupil was dilated under atropia, but the ophthalmoscope showed the fundus to be clouded with blood. The patient had no pain. Vision equaled No. 5 Snellen, at two meters.

My diagnosis was steel in the vitreous humor. Under cocaine I introduced a point of the electro-magnet through the wound, and at a depth of not more than three millimeters, caught a piece of iron and drew it out by a little coaxing without the aid of any other instrument. The conjunctiva was drawn together over the sclerotic wound, a solution of atropia was instilled into the eye, and a bichloride dressing applied.

The steel was a thin scale, about seven millimeters long, one and one-half wide, and weighed one-half grain. The eye recovered without any inflammatory reaction, and, at a subsequent examination, the vision was found to be No. 5 Snellen, at five meters.

Case VIII.—Arthur S., aged 24, machinist, on January 14, 1892, while holding a mandrel for another man to strike with a sledge-hammer, a piece of something flew and struck his right eye. He consulted me about four hours after the injury, and I found a clean-cut, vertical wound at the outer margin of the cornea in the sclera. It was about three millimeters long, and the periphery of the iris was drawn into it, giving the pupil an irregular shape and a displaced position. With a probe, gently used, the iris and pupil were restored to their proper position, when an opening was detected through the iris and suspensory ligament of the lens. The eyeball was red, but the patient complained of very little pain. I could not see the bottom of the fundus with the ophthalmoscope on account of the blood in the vitreous. Vision equaled No. 9 Snellen, at one meter.

My diagnosis was a piece of iron in the vitreous humor, but on several careful trials, with various sized points of the electro-magnet introduced through the wound, I could not find any. Failing in these efforts, I instilled a six-grain solution of atropia and applied bandages, with directions to call again the next day.

January 15th, 10 a. m.—The eye had been comfortable since last visit. Vision equaled No. 9 Snellen, at one and a half meters. The pupil was dilated, and with the ophthalmoscope I could see at the upper and back part of the fundus, to the right side, a black opacity, appearing to be about six millimeters in diameter, making positive the diagnosis of steel in the vitreous chamber. The corneal wound had closed and the aqueous humor had reformed. Atropia was again instilled and the eye bandaged.

January 16th, the eye was more inflamed. Vision equaled No. 24 Snellen, at one meter. Dr. Abbott was called in consultation and agreed with me as to the diagnosis, and that another attempt at extraction should be made. After cocainizing the eye thoroughly, a small conjunctival flap was raised from the sclera on the outer side of the ball, between the external and inferior recti muscles. After the hemorrhage was stopped, a horizontal incision about four millimeters long was made through the sclera into the vitreous. Through this I introduced an extension-point of the electro-magnet, which I had curved somewhat, whose length was about one centimeter, and at its end, one and one-half millimeters in diameter. At a depth of about five millimeters the magnet caught the piece of iron, and it was easily brought out. It was a thin scale, two millimeters long, and one and one-half wide, and weighed about one-fourth of a grain. The conjunctival flap was replaced with a suture at its apex, atropia was instilled and the eye bandaged.

January 17th, 11 A. M.—He had very little pain during the past twenty-four hours. Pupil was well dilated and vision equaled No. 9 Snellen, at two meters. The eye afterwards healed well, but on March 22d, vision was lowered to a few of No. 12 Snellen, at two meters. The media were transparent.

CASE IX.—John S., aged 20, boiler-maker, consulted me February 21, 1892, with a history that two hours before, while driving a steel plug into a boiler, a piece of iron struck the left eye. There was a small wound through the lower lid below the central part of its margin, and one through the sclera a little to the left of the lower margin of the cornea. Beneath this a small, shiny spot could be seen in the iris. A fine probe showed that the wound extended into the vitreous at this point. Vision equaled No. 36 Snellen, at one meter. Under cocaine the electro-magnet was applied, introducing a small point one centimeter long through the original wound, which had been slightly enlarged with a knife. At the depth of about three millimeters the magnet caught the piece of steel, and after several attempts it was drawn out. Atropia was instilled and the eye was dressed antiseptically.

The steel was a thin scale about one and one-half millimeters square. The eye made a rapid recovery, and on May 29th, with $-3.00$ D, vision equaled No. 18 Snellen, at five meters, and the media were transparent.

CASE X.—W. T. G., aged 30, machinist, was brought to me by Dr. A. G. Bennett for consultation August 8, 1892. Six weeks before he was struck in the left eye with a piece of steel. Vision was impaired at once. The patient had a druggist look at his eye, but no foreign body could be seen. He gave him some "eyewater," which he used and the eye gave no pain or trouble. But, as the vision did not improve, he applied to Dr. Bennett a few days ago, who examined him, and made a diagnosis which I consider most commendatory to his judgment and skill as a young practitioner. My own examination simply confirmed his diagnosis.

Externally, the eye appeared perfectly normal, except that a minute hole could be seen in the iris at its inner part near its periphery. The pupil being dilated with a mydriatic, the ophthalmoscope showed a few minute opaque dots in the lens as if "peppered" in half a dozen places; but otherwise the media were transparent. Every part of the fundus was normal, except at the macular region. Here, extending from the macula lutea, directly upwards was a black line, in length a little less than the diameter of the optic disc, and in width a little more than the diameter of the largest retinal vein. It showed a slight luster, and on each side was seen a white line, evidently the sclera exposed through the split choroid. Around this was a line of pigment. Peripheral vision was apparently normal, but central vision, and that part of the field a little below the center, was lost. At a distance of five meters the central scotoma was about the size of a man's head. I did not take a perimetric chart of the field. By looking a little eccentrally, vision equaled No. 60 Snellen, at five meters. There was no doubt in my mind as to the correctness of Dr. Bennett's diagnosis of steel in the retina, at the upper part of the macula lutea.

What should be done? It was agreed that the eye would be better off with the steel out, that very little danger would attend a careful attempt at extraction, and that if the effort was unsuccessful, the eye would not suffer more in the end.

The eye, therefore, was thoroughly anesthetised with cocaine and the proposed operation begun. A triangular conjunctival flap having been raised from the sclera in front of the equator, and between the external and inferior recti muscles, hemorrhage stopped, and a horizontal incision, five millimeters long, carried through the bared sclera into the vitreous by Dr. Bennett. I placed the patient in a convenient position in which I could use both the ophthalmoscope and the electro-magnet. I used an extension-point, one and one-half centimeters long, with end one millimeter in diameter, and curved so as to be directed easily towards the macula. With my right hand I introduced this point through the sclerotic incision, and under the clear observation of the fundus, with the ophthalmoscope held in my left hand, cautiously passed it through the vitreous to the macular region, lightly touched the black object, and then withdrew the magnet with the offending body on its point. The single attempt secured the coveted trohpy.

The conjunctival flap was replaced and held by a suture at its apex, atropia instilled, and the eye bandaged. No reaction followed the operation, and the eye rapidly recovered. The steel extracted was a minute scale, one and one-half millimeters long and three-fourths of a millimeter wide.

October 23, 1892.—Eleven weeks after the operation the eye appeared to be normal on external examination, and with the ophthalmoscope, the pupil being dilated with a mydriatic, the lens presented the same appearance as before the operation, the vitreous was transparent, and at the macular region, at the point from which the steel had been extracted, there was an area of about the same extent (although a little wider) as seen at the first examination, resembling in disturbance of pigment and color a patch of choroidal atrophy. Vision was the same as before the operation, but the eye was more sensitive to light.

This case, to me, is a most interesting one, and is one among a very few on record in which steel has been successfully extracted from the retina. The injury which the steel inflicted was peculiar in view of its location, and although the vision was permanently impaired by it, the operation of extraction did not further lessen it.

Remarks.—In this group of ten cases, all that I have seen, it will be noted that in every case the steel was found in some part of the vitreous space. In every case it had entered near the corneo-scleral junction and passed through the iris and suspensory ligament or ciliary body. In one case it passed through the lower lid first, and then through the cornea and iris into the vitreous. In eight cases it seemed to be resting in the vitreous. In one it it was lodged in and held at its outer extremity by the ciliary body, and in one it was imbedded in the retina. The size of the steel varied from a minute scale to a large piece weighing seven grains. In every case but one the accident occurred while striking iron with a hammer, and the steel was propelled with great momentum. One eye was lost by infection, although antiseptic precautions were taken in this, as in all the other cases. In three cases the first attempt at extraction failed. In one of these a second attempt through the original wound, and in two through a sclerotic incision, was successful. In the first of these the eye became infected and was afterwards enucleated. The other two recovered with useful vision. In seven cases the steel was extracted through the original wound at the first attempt. In one the first attempt was by an incision through the sclera and was successful.

These cases only add further and impressive illustrations to those furnished by other ophthalmic practitioners of the benefits of the electro-magnet in the class of injuries under consideration. The lesson is that with it a great majority of the cases in which iron has been driven into the cavities of the eye, this can be saved with more or less vision, while without it almost every one would be totally lost, and perhaps cause, also, the loss of its fellow eye. In some cases the injury to an eye is so great that no attempt should be made to save it by removing the steel, but it should be enucleated or eviscerated at once.

In considering the propriety or need of using the electro-magnet, a correct diagnosis is always desirable, but this is often not easily arrived at.

The history of the case that in hammering or working iron a splinter has struck the eye, that it has made a wound through its coats, and that the piece was not afterwards found by the patient, makes the presumption very strong that it has entered the eye. As a rule, a minute object striking the eye with sufficient force to cut through its coats is carried on into the non-resisting fluids within by its own momentum. The examination of the eye by oblique illumination and the ophthalmoscope, the pupil being dilated, if possible, sometimes gives negative and sometimes positive information.

Steel may be lodged at any point in the cornea, sclera, iris, lens, or in the vitreous or inner coats of the eye. Blood and inflammatory exudates and opacities of the cornea or lens obscure the view. But when there is no obscuration, steel is easily recognized by its black appearance, or by its shining luster from the reflection of the light from the ophthalmoscope or focal illumination. This lustrous reflection often replaces the dark appearance of the iron. If the iron is in the vitreous cavity, it will appear, when seen with the ophthalmoscope, very much magnified in size.

The sensations of the patient or the state of vision are of very little value in making out a diagnosis. The diagnosis is positive when the foreign body can be seen; when it cannot be seen it is probable, in different degrees of certainty according to the history of the injury and the character of the wound inflicted.

In using the electro-magnet in a case in which steel has either, probably or positively entered the eyeball, it should be made to pass as directly as possible to the supposed or known location of it in lines of least resistance, and through an area of least functional value. Sometimes the accident-wound meets these requirements, but oftentimes it does not. If steel has penetrated into the vitreous through the cornea near its margin and through the iris and suspensory ligament of the lens, there is always great danger of adding further traumatism to the parts, and especially to the lens. That part of the ball through which the magnet can be introduced with least danger to important structures and to sight, and, at the same time, most accessible to every point within the vitreous chamber, is the sclera just in front of the equator of the ball, and preferably in most cases on the outer side between the external and inferior recti muscles. It is better to make the sclerotic incision after dissecting up, at the place chosen, a small triangular flap of conjunctiva, or excising a piece and stopping the hemorrhage.

Had I selected this position for operative procedure in Case V., instead of endeavoring to follow the track of the steel, I have no doubt that I would have found and removed it on the first attempt and saved the eye.

In Cases I. and VIII. the effort to extract the steel through the corneal and iris wound failed after several trials. But on opening the sclera, as described above, the first introduction of the magnet secured and brought it out.

Case X. shows, also, how the magnet-points may be passed directly to the macular region (and, indeed, to any other,) with the minimum of operative traumatism to the eye. It is also a forcible illustration of the greater ease and safety with which a piece of steel imbedded in the coats of the eye posteriorly can be reached than by more extended operative procedures.

The early use of the electro-magnet is preferable to waiting till pathological processes follow the introduction of the steel. Sometimes, it is true, the eye will tolerate steel for an indefinite period of time, perhaps for years, but this is the exception rather than the rule. By waiting, the steel becomes embedded in exudates, when the magnet cannot attract it at all, and if the eye does become inflamed, enucleation is then the only safe treatment. The proper use of the magnet is usually so successful when applied early, that it seems to me there is no alternative between immediate operation and delay.

The form of electro-magnet, especially of the extension-points, is a matter of no little importance. The magnet itself should be as light as possible consistent with sufficient attracting power, and of a shape convenient for handling. The extension-points should be as near as practicable to the coil around the core, and no longer than is necessary to reach the supposed location of the steel, and certainly never more than two centimeters ($\frac{3}{4}$ of an inch), and at the ends from one-half to one and one-half millimeters in diameter.

The power of attraction diminishes very rapidly as the end is lessened in size or carried further from the coil around the core. Again, the points should not be rounded, but flat at the ends, and also on the sides from the ends backwards for a short distance, as by this a larger surface of contact is presented and the holding power increased. The magnet which has served me so well is constructed upon these principles.

In conclusion, I submit this report to emphasize anew the utility and benefits of the electro-magnet in ophthalmic surgery; to recommend its early employment; to call attention to a special form of instrument which, I believe, better than some others, fulfills the requirements needed; and in addition, to advocate a more general adoption of the sclerotic incision, as the safest method of reaching steel when it lies at any point, supposed or known, in the vitreous humor, or the inner coats of the eye, even to the disregard of the original wound in many cases.

212 Franklin Street.

